# Enhancing mechanical properties of flash-spun filaments by pressure-induced phase separation control in supercritical high-density polyethylene solution

**DOI:** 10.1038/s41598-022-22781-1

**Published:** 2022-10-27

**Authors:** Jae-Hyung Wee, Younghwan Bae, Nam Pil Cho, Moo Sung Kim, Won Jun Lee, Sang Young Yeo

**Affiliations:** 1 Advanced Textile R&D Department, Korea Institute of Industrial Technology, 143 Hanggaulro, Sangnok-Gu, Ansan-Si, Gyeonggi-Do 15588 Republic of Korea; 2 Department of Fiber System Engineering, Dankook University, 152 Jukjeon-Ro, Suji-Gu, Yongin-Si, Gyeonggi-Do 16890 Republic of Korea; 3 Department of Polymer Engineering, Graduate School, School of Polymer Science and Engineering & Alan G. MacDiarmid Energy Research Institute, Chonnam National University, 77 Yongbong-Ro, Buk-Gu, Gwangju, 61186 Republic of Korea

**Keywords:** Engineering, Materials science

## Abstract

Flash-spun nonwoven (FS-NW) is gaining attention in the PPE field due to its excellent barrier and mechanical properties resulting from its non-uniform diameter distribution and unique filament morphology. The unique network structure of flash-spun filaments (FSF) comprising the FS-NW can be controlled by phase separation behavior in the supercritical fluid (SCF) process. This study proposes a simple method to control the microstructure of FSFs by controlling the pressure-induced phase separation (PIPS) process in polymer/SCF solution. This phase separation behavior of an HDPE/SCF solution was confirmed by using a high-pressure view cell. A multistage nozzle allowing for phase-separated pressure to form different phases was also designed. HDPE-FSFs were synthesized by flash-spinning, and their morphology, crystallinity, and mechanical properties were investigated. The results demonstrated that the filaments obtained by PSP control at 220 °C and with an HDPE concentration of 8 wt% showed a network structure composed of strands, wherein the diameters ranged from 1.39 to 40.9 μm. Optimal FSF was obtained at 76 bar, with a crystallinity of 64.0% and a tenacity of 2.88 g/d. The PIPS method can thus effectively control the microstructure more feasibly than temperature- or solvent-induced techniques and can allow the effective synthesis of various products.

## Introduction

The safety and wellness of people in modern society are vulnerable to factors that threaten the human body, such as severe air pollution, pathogens, and viruses. The novel coronavirus disease (COVID-19) is a striking example of this phenomenon as it has caused a global pandemic since it was first observed in 2019 and continues to exact a significant human toll^1,2^. Viruses are typically known to spread through small aerosols (usually defined as < 5 µm), or larger respiratory droplets expelled when coughing, sneezing, or breathing^3,4^. Therefore, the development of personal protective equipment (PPE) to prevent the spread of infection, and to protect both patients and medical workers from dangerous exposure is gaining increasing importance.

Generally, PPE is worn to minimize exposure to hazards that can cause serious workplace injury and illness, and may include items from gloves and safety glasses to shoes, earplugs, hard hats, respirators, and full-body suits^5–7^. PPE material requisites certain characteristics such as considerable mechanical/structural strength that can stand for strenuous activity, barrier properties against the external environment, and filtration of pollutants^6,7^. Among the materials that are used to construct PPE, micro/nanofiber nonwoven is currently very popular as an essential constituent of respiratory or full-body protective equipment. Micro/nanofiber nonwovens have a high filtration efficiency owing to several advantageous properties such as small fiber diameter, large surface area to volume ratio, high porosity, and good internal connectivity^6,8–10^. These nonwovens are generally obtained via widely practiced spun-bond or melt-blown processes that allow for excellent air permeability and filtration efficiency. However, it is challenging to obtain products with mechanical strength capable of handling vigorous human activity via these methods.

Flash-spun nonwoven (FS-NW) fabric is attracting attention as a promising PPE material owing to its excellent functional traits such as high tensile and tear strength and moisture-permeable waterproof properties^7,11^. FS-NW fabric consists of microfibers with a diameter distribution ranging from tens of micrometers to hundreds of nanometers, resulting in higher tensile and tear strength than typical spun-bond nonwoven fabric with a fiber diameter of ≥ 10 μm, and barrier properties comparable to those of polymer membranes^11–13^. The network filament morphology, attributed to the flash-spinning process, allows for these unique properties of FS-NW. Flash-spinning is a high-end process for the production melt-spun nonwoven fabric, utilizing a supercritical fluid (SCF) process^12,14–16^. SCFs can be used as highly effective media in polymer processing as they exhibit liquid-like density and solubility while also possessing gas-like transport properties. Additionally, the phase behavior of their solutions can easily and conveniently be controlled by changes in temperature and pressure^17^. In the flash-spinning process, a polymer is dissolved in a high-pressure and temperature (HPT) SCF and then spun via instantaneous ejection at normal pressure and temperature (NPT)^12,15,16,18^. Prepared by spontaneous pressure while heating the polymer–solvent mixture, this single-phase polymer/SCF solution separates by a decrease in pressure and subsequently ejects through an orifice into a substantially lower pressure and temperature (usually NPT) region to form the FSF^12,16,18^. Phase separation in the SCF mixture during this procedure can lead to profound structural changes in the flash-spinning filaments (FSF), the extents of which depend on the process parameters, such as temperature, pressure, and concentration. Although studies on phase separation behavior in polymer/SCF solutions are being conducted^19–21^, it is difficult to apply the research approach to the actual flash-spinning process, so systematic studies on the effect of phase behavior on the material properties of the resulting product are insufficient.

In this study, we prepared a polymer/SCF solution using trichlorofluoromethane as the solvent and high-density polyethylene (HDPE) as the fiber precursor, and performed flash-spinning by pressure-induced phase separation (PIPS). To use the polymer/SCF solution in the flash-spinning, the phase separation is inevitably accompanied. PIPS is advantageous in that pressure changes can be provided as an experimental uniformly control parameter across the entire polymer/SCF system^22^. In this work, we observed the phase behavior of the HDPE/SCF solution relative to pressure changes made to control the PIPS process. Based on this phase-separated pressure (PSP), a multistage nozzle with a region to enforce a pressure drop was designed and applied to flash spinning. Finally, the effect of PIPS on the morphological, crystallographic, and mechanical properties of FSFs was investigated.

## Results and discussion

**Figure 1:**
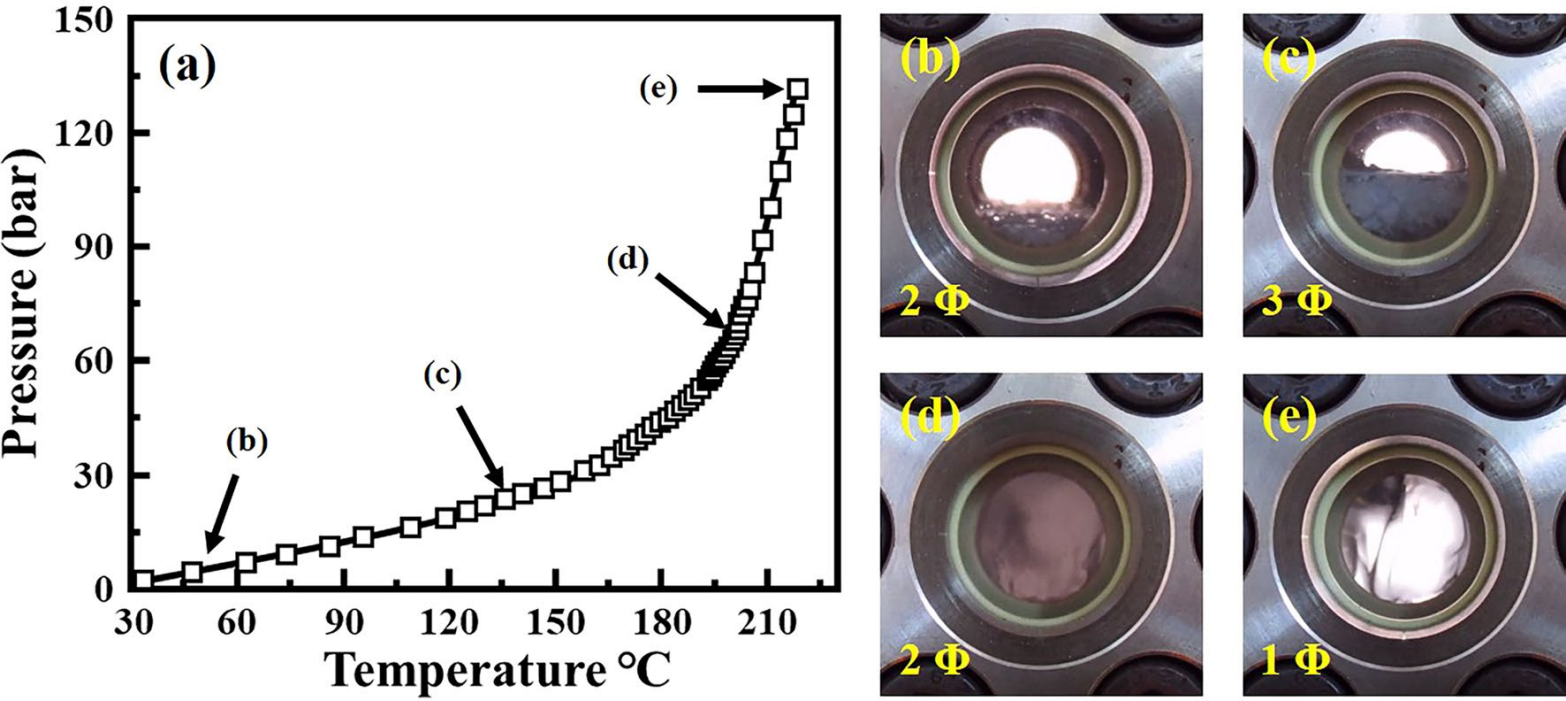
Pressure–temperature variation of the HDPE/solvent mixture. (**a**) Profile of pressure variations up to 220 °C and (**b**–**e**) optic images at the phase change in the high-pressure view cell; (**b**) two-phase (51 °C/3.4 bar), (**c**) three-phase (134 °C/18.1 bar), (**d**) two-phase (198 °C/38.8 bar), and (**e**) single-phase (220 °C/134 bar).

The formation of the HDPE/SCF solution was visually confirmed by the apparent phase change between the HDPE and solvent, as observed via the high-pressure view cell. Figure 1a shows the pressure change in the vessel and phase changes of HDPE and solvent against temperature increase up to 220 °C. The solvent is seen to undergo vaporization as the temperature rises, condensing under spontaneous pressure and mixing with the molten HDPE. Thus it can be confirmed that the polymer/solvent mixture above the critical point (spontaneous pressure is 134 bar at 220 °C) forms a polymer/near-SCF solution. To process polymers from the SCF solution, information on the phase behavior of the polymer/SCF solution is required. The S–L–V (Solid, Liquid, and Vapor, respectively) mixed phase formed due to the vaporization of the solvent from the S-L mixed phase (Fig. 1b), with increasing the temperature. This was observed as the solvent is a light component (Fig. 1c). Following this, the polymer melted above T_m_, resulting in an L phase, and the vaporized solvent was condensed by the spontaneous pressure rise, thereby giving rise to the L–L phase (Fig. 1d). Finally, the L–L mixed phase crossed the critical point to form a single-phase (L) (Fig. 1e). From these phase behaviors, it can be stated that for the composition of the HDPE/solvent used and the specific molecular weight of the HDPE, the system follows the generally known HPT phase behavior in the polymer/SCF solution^19,20,23^.

**Figure 2:**
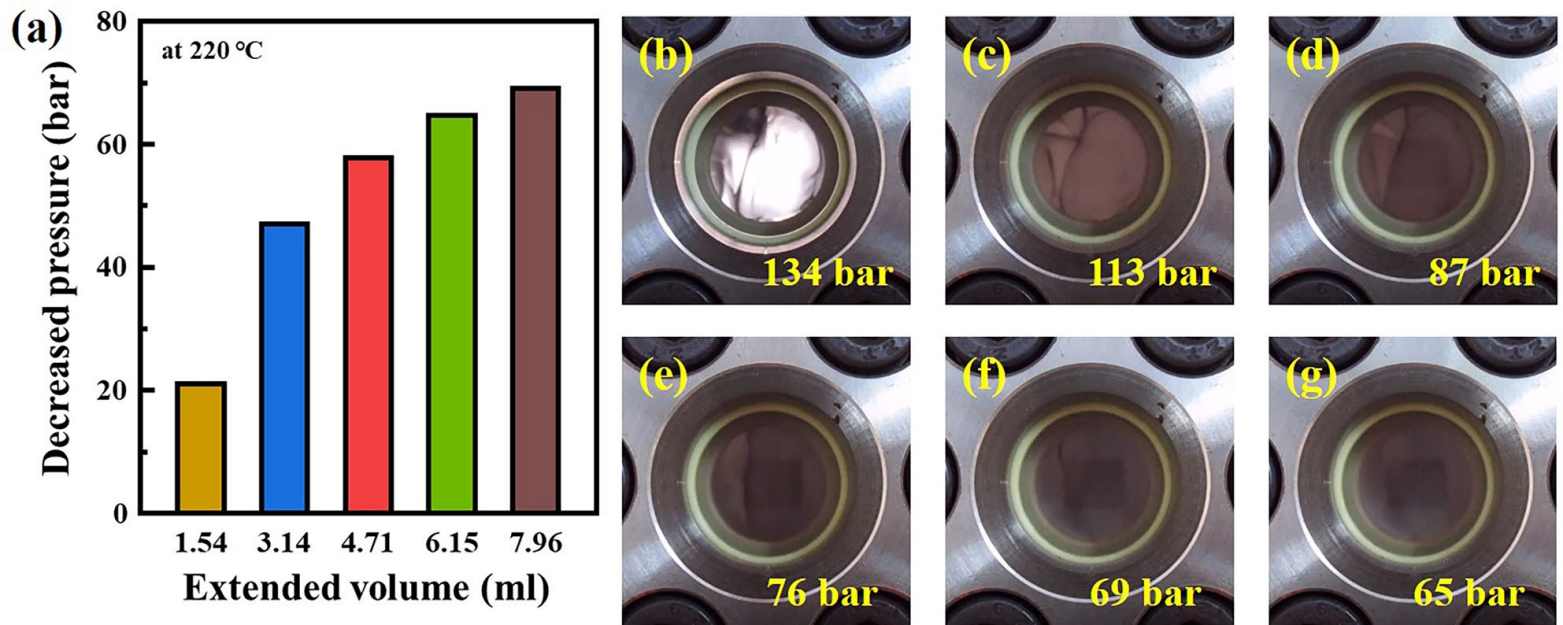
Dropped pressure against the extended volume of the HDPE/SCF solution and the optic images of phase changes at each reduced pressure. (**a**) Pressure–volume relationship and phases at: (**b**) 134 bar, (**c**) 113 bar, (**d**) 87 bar, (**e**) 76 bar, (**f**) 69 bar, and (**e**) 65 bar (the initial pressure: 134 bar).

The pressure-induced phase behavior of the HDPE/SCF solution at 220 °C was observed by changing the internal volume of the high-pressure view cell by adjusting of the piston. Figure 2 shows the dropped pressure due to the extended internal volume and the optic images of the phase changes at each pressure. The pressure of the HDPE/SCF solution was observed to decrease as the internal volume extended, as shown in Fig. 2a. With this decrease in pressure, the transparent phase gradually became cloudy (Fig. 2c,d). Pressure drops above 60 bar completely darkened the phase, and the phases at 69 bar and 65 bar are difficult to distinguish between (Fig. 2e–g). This phase change is attributed to the phase separation of the polymer phase and the solution phase as the pressure of the HDPE/SCF solution decreases; the decrease in pressure reduces the density of the solvent, which in turn reduces the solubility of the polymer, ultimately causing phase separation^20,21,23^. This phase response to changing pressure suggests that different phase separations can be induced in the HDPE/SCF solution simply through pressure control.

**Figure 3:**
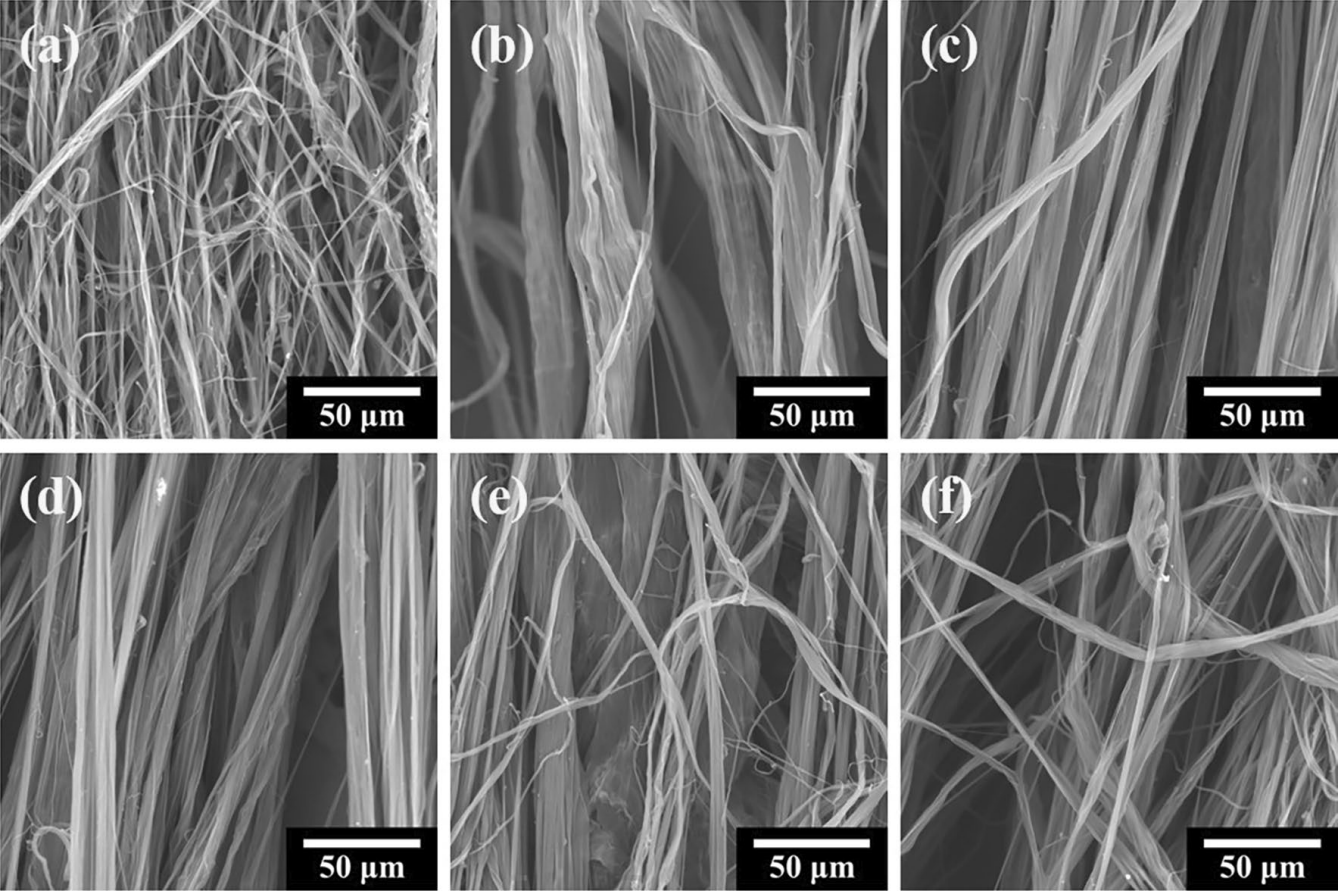
Scanning electron microscope images of FSFs obtained at different PSP values. Images corresponding to (**a**) PSP-134, (**b**) PSP-113, (**c**) PSP-87, (**d**) PSP-76, (**e**) PSP-69, and (**f**) PSP-65.

**Figure 4:**
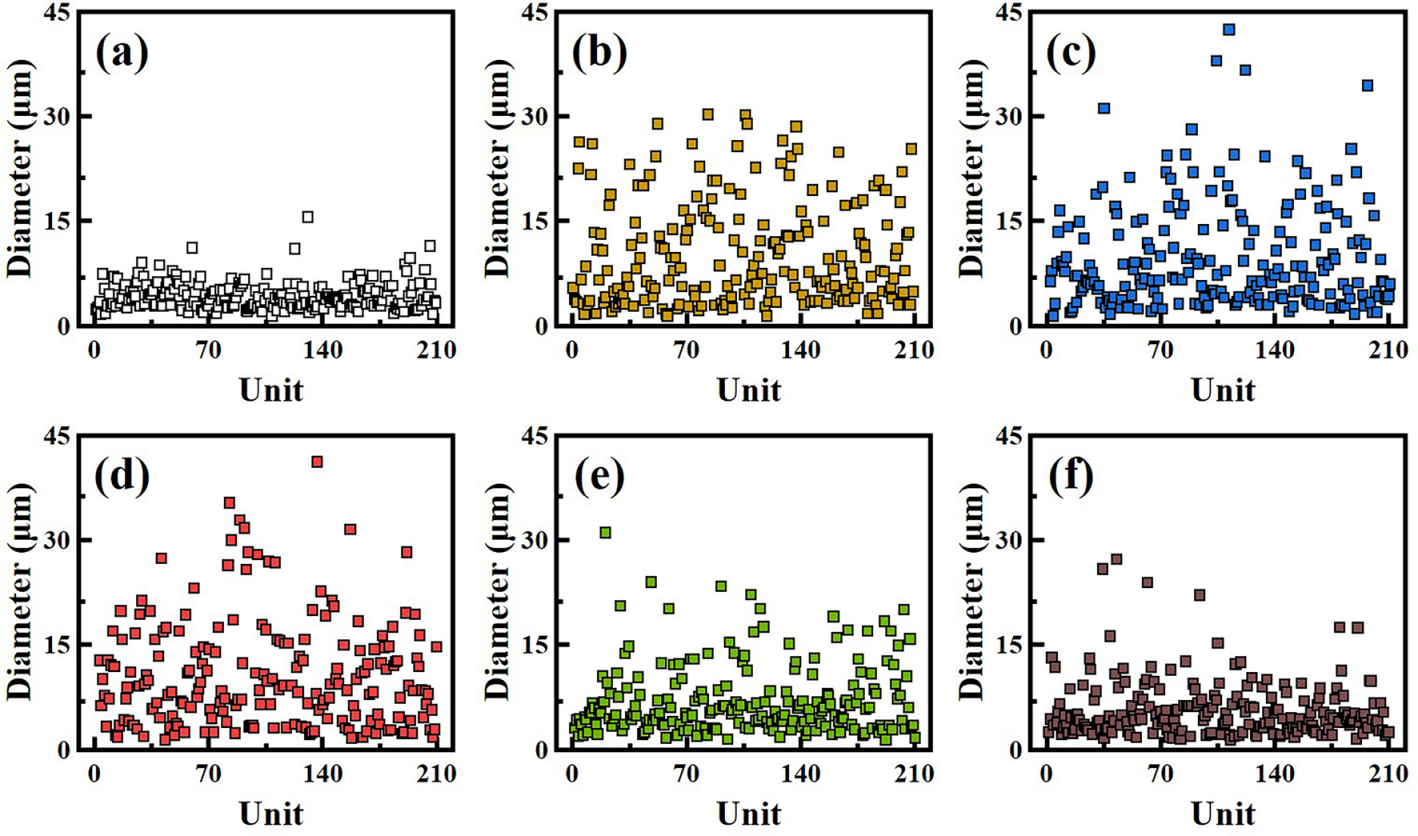
FSF strand diameter distributions obtained at different PSP values. Distributions for (**a**) PSP-134, (**b**) PSP-113, (**c**) PSP-87, (**d**) PSP-76, (**e**) PSP-69, and (**f**) PSP-65.

The flash-spinning solution was prepared under the same conditions as those observed in the high-pressure view cell. The FSFs obtained by flash-spinning were named according to the phase-separated pressure (PSP). That is, the samples are denoted as PSP-134, -113, -87, -76, -69, and -65. Figure 3 shows scanning electron microscope (SEM) images of the FSFs obtained from the corresponding PSPs in Fig. 2. Interestingly, unlike the single filament obtained by using a single-hole nozzle such as in conventional melt spinning, FSFs exhibit a net-like morphology consisting of numerous strands. This non-uniform filament morphology with varying strand diameters is known as film-fibril plexifilament^12,16,18^. In particular, it can be seen that the diameter of the strands increased at lower PSP values, but the strands became thinner again at the relatively low PSP value of 69 bar. The diameter of 200 strands was measured from each SEM image to analyze the variation in the distribution of strand diameter in the obtained FSF (Fig. 4). Strand diameter distribution is closely related to the barrier properties of FS-NW fabric. Strands of various thicknesses increase the packing density of the nonwoven fabric to improve the barrier properties. The PSP-134 without PSP control had a narrow distribution of strand diameters from a minimum of 1.39 μm to a maximum of 15.5 μm with a variance value of 4.1 (Fig. 4a). As for lower PSP values, the strand diameter distribution of PSP-76 was found to be broad, from a minimum of 1.16 μm to a maximum of 40.9 μm, and showed a variance of 65.3, the highest of all the samples (Fig. 4d). PSP-65, the lowest PSP value, had small strand diameters from a minimum of 1.33 μm to a maximum of 27.1 μm and exhibited a low dispersion value of 17.3 (Fig. 4f). These strand diameter and distribution variations can be attributed to the phase separation pathway of the polymer/SCF solution.

**Figure 5:**
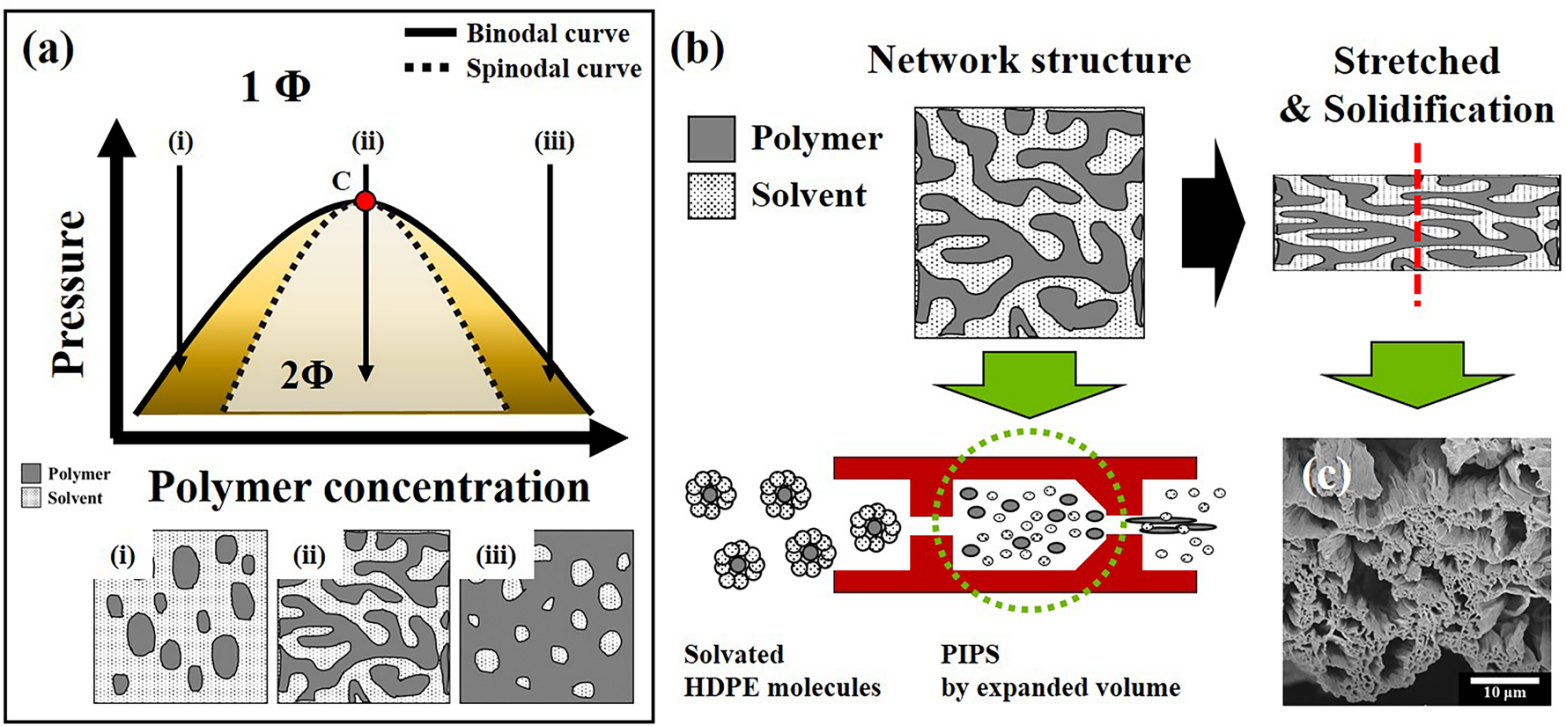
Representations of phase separation pathways and network structure formation. (**a**) Schematics of phase separation pathways in polymer/SCF solution, (**b**) network structure formation in the FSF by phase separation at the flash-spinning process, and (**c**) cross-sectional SEM image of FSF-76.

Under constant temperature, the polymer/SCF solution undergoes a PIPS pathway that passes through the following regions; a stable region which exists as a single phase, a metastable region in which two phases coexist, and an unstable region where phases are completely separated (as shown in Fig. 5a)^22,24,25^. In this typical phase separation pathway, the metastable region is observed when molecules gather to begin nucleation, followed by growth, ultimately resulting in phase separation^17,20,22^. In this system, the spinodal decomposition mechanism refers to the phase separation that barely passes through the metastable region. The separated polymer and solvent phases have three types of solid forms depending on the fraction of polymer and solvent; (i) discontinuous solvent-rich polymer phase (particle)^26,27^, (ii) continuous polymer/solvent phase in which the polymer and solvent are produced simultaneously (network)^24,28–30^, and (iii) discontinuous polymer-rich solvent phase (porous)^31,32^. In our particular case, the FSF morphologies observed in Fig. 3 suggest that the polymer fraction in the prepared HDPE/SCF solution undergoes the separated phase of (ii). In the instantaneous phase, the separated HDPE/solvent mixture is stretched by the ejecting pressure and solidified via rapid cooling from the expansion and vaporization of the solvent. Ultimately, a three-dimensional filament consisting of strands with porous cross-sections is obtained, as shown in Fig. 5b and c. We controlled the phase separation point of the PIPS pathway by the specific PSPs, as confirmed in Fig. 2a. PSP-76 exhibited a broad strand diameter distribution, which suggests sufficient growth of polymer nuclei in the metastable region of the PIPS pathway. On the other hand, FSFs obtained from the early and late phases in the PIPS pathways (excluding PSP-76), that barely stay in the metastable region exhibit relatively narrow diameter distribution (Fig. 4). Thus, these results indicate that the pressure controlled phase separation point at the PIPS significantly affects the intrinsic morphology of the FSFs obtained and alters the distribution of strands diameter.

**Figure 6:**
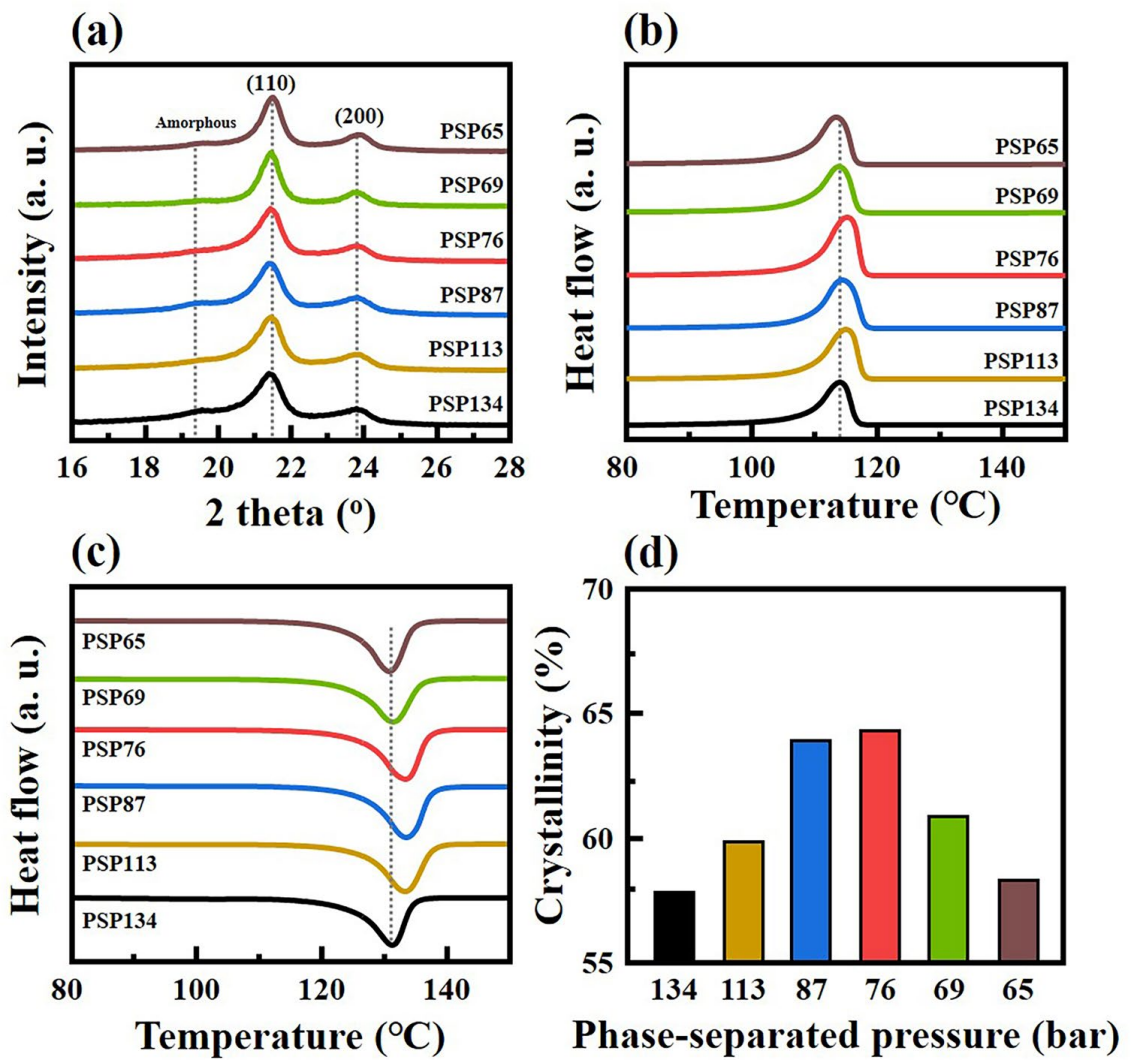
Characterization results for FSFs obtained at different PSPs. (**a**) XRD patterns, (**b**, **c**) DSC curves, and (**d**) crystallinity of FSFs obtained via DSC results.

The X-ray diffraction (XRD) pattern for the FSF samples is shown in Fig. 6a. XRD patterns of the FSFs mainly show two peaks at 21.5° and 23.9°, which were identified as the (110) and (200) planes of HDPE, respectively^33,34^. These data further indicate that FSFs have an orthorhombic structure^35^. Some of the other small peak confirm the semi-crystalline nature of the HDPE (i.e. the presence of crystalline and amorphous regions). A broad shoulder peak at about 19.3° is observed next to the (110) peak in PSP-134, which means that PSP-134 has an amorphous structure of HDPE. The shoulder peak gradually decreased with decreasing PSP, suggesting a decrease in the amorphous region (as seen in PSP-76). However, in the case of PSP-65, the shoulder peak increases, suggesting that an amorphous region increases in this sample. Figure 6b and c show differential scanning calorimetry (DSC) curves of the FSF samples, and thermal properties obtained via DSC are summarized in Table S1. The DSC behavior of FSF demonstrated the tendencies of a typical semi-crystalline polymer. Depending on the PSP, the FSF samples were seen to exhibit different melting and crystallization temperatures (T_m_ and T_c_). PSP-134 was found to have a T_m_ and T_c_ of 131.3 °C and 114.1 °C, respectively, whereas PSP-76, which was phase separated at relatively low pressures, exhibited a T_m_ and T_c_ of 133.3 °C and 115.2 °C, respectively. It is apparent that these parameters were slightly higher for the latter. On the other hand, PSP-65, which phase-separated at the lowest pressure, showed a lower T_m_ and T_c_ as compared to PSP-134, at 130.7 °C and 113.4 °C, respectively. This shift in T_m_ and T_c_ suggests that the crystals formed by PSP in FSF differ. Figure 6d shows that the crystallization enthalpy (∆H_c_) of PSP-134 was found to be 165.9 J/g, which indicates a crystallinity of 57.6% based on 100% crystallized HDPE (288 J/g)^36^. The lower the PSP, the higher the ∆H_c_, and accordingly, PSP-76 showed the highest ∆H_c_ of 184.4 J/g with a 64.0% degree of crystallinity. On the other hand, PSP-65 had a ∆H_c_ of 167.5 J/g, showing crystallinity of 58.3%, similar to that of PSP-134. These results are also similar to the relative crystallinity obtained from the XRD results. These XRD and DSC results suggest that PSP affected nucleation and growth of polymers solvated by supercritical fluids during phase separation^22,37^. As mentioned above in relation to the phase separation pathway, PSP-134 without PSP control forms polymer nuclei in the metastable region but rapidly destabilized before growing, resulting in low crystallinity. Additionally, even at the lowest PSP (65 bar), the rapid phase separation before ejection causes nucleation and the ejection of polymer nuclei into a stationary phase, resulting in low crystallinity. On the other hand, the data obtained supports that if a suitable PSP is observed, the polymer nuclei formed in the metastable region experience sufficient growth before the ejection, thereby achieving a high degree of crystallinity. Moreover, a change in crystallinity is closely related to the trends observed in the distribution of strand diameter. As the degree of crystallinity increases, the larger diameter strands increase, resulting in a wider distribution (as shown in Fig. 4). This trend suggests that the growth of polymer nuclei corresponds to the development of the network structure. In the filament formation step, it can be seen that the variation in the PSP leads to the formation of different network structures, and consequently, variation in the distribution of strand diameter.

**Figure 7:**
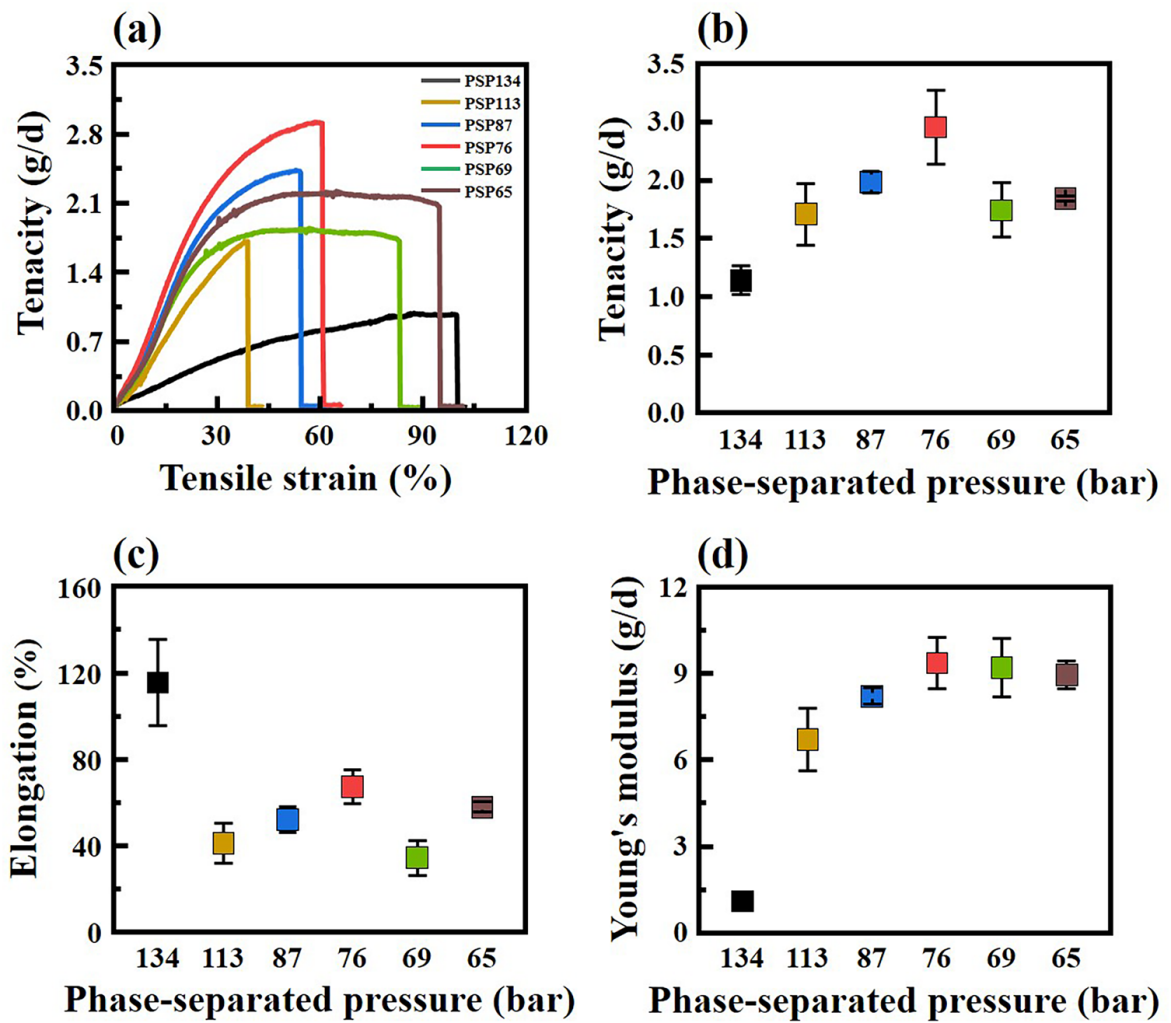
Mechanical properties of FSFs obtained at different PSPs. (**a**) strain–stress curves, (**b**) tenacity, (**c**) elongation, and (**d**) Young’s modulus.

The mechanical properties of FSFs are crucial for the applications of nonwoven. High tear strength and stiffness of FS-NW can be ascertained from the mechanical properties of the FSFs. The mechanical properties of the obtained FSFs are shown in Fig. 7. PSP-76 showed the highest tensile strength of 2.88 g/d, whereas FSFs obtained from PSPs of 67 bar and lower showed a relative decrease in tensile strength. The Young’s modulus of the FSFs gradually increased as PSP decreased. The elongation decreased rapidly in PSP-113 and increased as the PSP decreased, which is attributed to the degree of growth of the polymer nuclei due to phase separation. It is well known that the mechanical properties of a material are generally affected by crystallinity of polymer or orientation of polymer chain^12,14^. We confirmed the orientation of the polymer crystals of FSF from an azimuthal scan using wide-angle x-ray diffraction (WAXD) (Fig. S1). The preferred orientation of FSF-134 without using multi-stage nozzles was 66.6%, which was low. On the other hand, all FSFs obtained using multi-stage nozzles showed a preferred orientation of over 80%, and FSF-76 had the highest orientation of 88.9%. FSF-69 and FSF-65 showed slightly decreased directionality. These results indicate that PSP significantly affects the polymer chain orientation and mechanical properties. The change in tenacity of the FSF obtained through PSP control shows a trend similar to that of the correlation between strand diameter distribution and percentage crystallinity. Therefore, these results suggest that the crystallinity or network structure obtained by PSP control in the PIPS process is directly related to the mechanical properties of the obtained FSF. Moreover, the high tensile strength and a broad strand diameter distribution of PSP-76 by optimal phase separation can enhance the barrier and mechanical properties of FS-NW.

## Conclusions

In this work, we prepared a polymer/SCF solution and observed the relevant pressure-induced phase changes. It was confirmed that the polymer/SCF solution undergoes phase separation by the induced pressure drop, and exhibits different phases under controlled PSP. We obtained the FSF by flash-spinning at the observed PSPs via a multistage nozzle especially designed for this purpose. FSFs obtained by PIPS showed an intrinsic filamentary morphology composed of strands and had a different strand diameter distribution in relation to the controlled PSP. The FSF obtained from PSP at 76 bar showed the highest variance in strand diameter distribution, and also demonstrated the highest crystallinity: 64.0%. PSP influenced not only these traits but also the mechanical properties of FSF, with PSP-76 showing the highest tensile strength and Young's modulus at 2.88 g/d and 9.35 g/d, respectively. Therefore, we were able to investigate and obtain optimal physical properties of FSF by PIPS process in a polymer/SCF solution. It should be noted that our results were concerned with the flash-spinning process, and therefore only apply to a specific fraction. The crystallinity and network structure formation of FSFs prepared by the PIPS process for different polymer fractions will not be similar to our results. Furthermore, additional FS-NW related studies are currently underway, along with several other characterizations of FSFs obtained by PIPS.

## Methods

### Materials

HDPE for flash-spinning had a melting temperature (T_m_) of 135 °C, a melt index of 4.7 at 190 °C, and a density of 0.965 g/cm^3^. Trichlorofluoromethane was used as a solvent following two filtrations via microfilter. The boiling temperature of trichlorofluoromethane was 23.8 °C and the critical temperature (T_Cr_) and critical pressure (P_Cr_) were 197.9 °C and 43.9 bar, respectively.

### Flash-spinning apparatus and process

The lab-scale flash-spinning apparatus used consisted of a high-pressure vessel, a multistage nozzle, a drain pipe, and a high-pressure N_2_ accumulator (a schematic of the flash-spinning apparatus is shown in Fig. S2a). The multistage nozzle consisted of a primary nozzle (input to the pressure drop region), a secondary nozzle (output to NPT), and a volume to allow for pressure drop between them. Both primary and secondary nozzles were single-hole nozzles with a diameter of 0.7 mm (a schematic of the multistage nozzle is shown in Fig. S2b). Dope was prepared for spinning by mixing the trichlorofluoromethane of 568 g and 8 wt% of HDPE. The volume of the mixture is 87% of the volume of the high pressure vessel with 500 ml. The closed vessel was then heated to 220 °C at a rate of 5 °C/min while being stirred at 300 rpm. Subsequently, the HDPE/SCF solution was spun out by ejecting it to NPT through the multistage nozzle; the decompression of the vessel due to this ejection was compensated by the action of the high-pressure N_2_ accumulator.

### Characterization

A high-pressure view cell system capable of internal observation was used to evaluate the solubility of HDPE in SCF. The high-pressure view cell consists of a sight glass in a vessel containing about 85 ml of liquid and a piston for pressure control so that the pressure and phase change of the internal fluid can be observed in real-time as the temperature increased (as shown in Fig. S3). The morphology and diameter distribution of the FSF fiber was observed by SEM. The crystallographic structure and the thermal properties of the FSF were characterized via XRD analysis using Cu-Kα radiation, and DSC at a heating rate of 20 °C/min, respectively. To calculate the crystallinity of the FSF, the crystallization enthalpy (∆H_c_) was obtained by integrating the area under the melting curve of the DSC. Azimuthal scan was performed using WAXD to confirm the orientation of the polymer chain of FSFs. The mechanical properties of the FSF were determined using a universal tensile testing machine. Fiber samples were prepared for this measurement by twisting 10 times per inch^16,18^.

## Supplementary Information

Supplementary Information.

## Data Availability

The datasets used and/or analyzed during the current study are available from the corresponding author upon reasonable request.
