# Supplementary material for: Enhancing mechanical properties of flash-spun filaments by pressure-induced phase separation control in supercritical high-density polyethylene solution
Source: Sci Rep. 2022 Oct 27;12:18030. doi: 10.1038/s41598-022-22781-1 (PMC9607727; doi:10.1038/s41598-022-22781-1)
Supplement: Supplementary file 1 — Supplementary Information. [file 41598_2022_22781_MOESM1_ESM.docx]

***Supporting information***

**Enhancing mechanical properties of flash-spun filaments by pressure-induced phase separation control in supercritical high-density polyethylene solution**

Jae-Hyung Wee ^1†^, Younghwan Bae^1^, Nam Pil Cho^1,2^_,_ Moo Sung Kim^3^, Won Jun Lee^2^, Sang Young Yeo^1*^

^1^ Advanced Textile R&D Department, Korea Institute of Industrial Technology, 143 Hanggaulro, Sangnok-gu, Ansan-si, Gyeonggi- do, 15588, Republic of Korea

^2^Department of Fiber System Engineering, Dankook University, 152 Jukjeon-ro, Suji-gu, Yongin-si, Gyeonggi-do, 16890, Republic of Korea

^3^Department of Polymer Engineering, Graduate School, School of Polymer Science and Engineering & Alan G. MacDiarmid Energy Research Institute, Chonnam National University, 77 Yongbong-ro, Buk-gu, Gwangju 61186, Republic of Korea

* **Corresponding author**: miracle@kitech.re.kr (S. Y. Yeo)

**Table S1**. Thermal property parameters obtained by DSC curve at 20 ºC/min and relative crystallinity obtained by XRD of FSFs obtained at different PSPs.

| Samples | T_m_ ^a)^  (°C) | T_c_ ^b)^  (°C) | ∆H_c_ ^c)^  (J/g) | Crystallinity ^d)^  (%) | Crystallinity ^e)^  (%) |
| --- | --- | --- | --- | --- | --- |
| FSF-134 | 131.3 | 114.1 | 165.9 | 57.6 | 59.5 |
| FSF-113 | 133.2 | 115.1 | 171.7 | 59.6 | 63.9 |
| FSF-87 | 133.5 | 114.4 | 183.3 | 63.6 | 69.2 |
| FSF-76 | 133.3 | 115.2 | 184.4 | 64.0 | 73.3 |
| FSF-69 | 131.3 | 114.0 | 174.6 | 60.6 | 66.4 |
| FSF-65 | 130.7 | 113.4 | 167.5 | 58.1 | 64.8 |

*a) T_m_: Melting temperature, b) T_c_: crystallization temperature, c) ∆H_c_: crystallization enthalpy, d) crystallinity calculated based on 100 % crystallization of HDPE (288 J/g), e) relative crystallinity obtained by XRD.

**
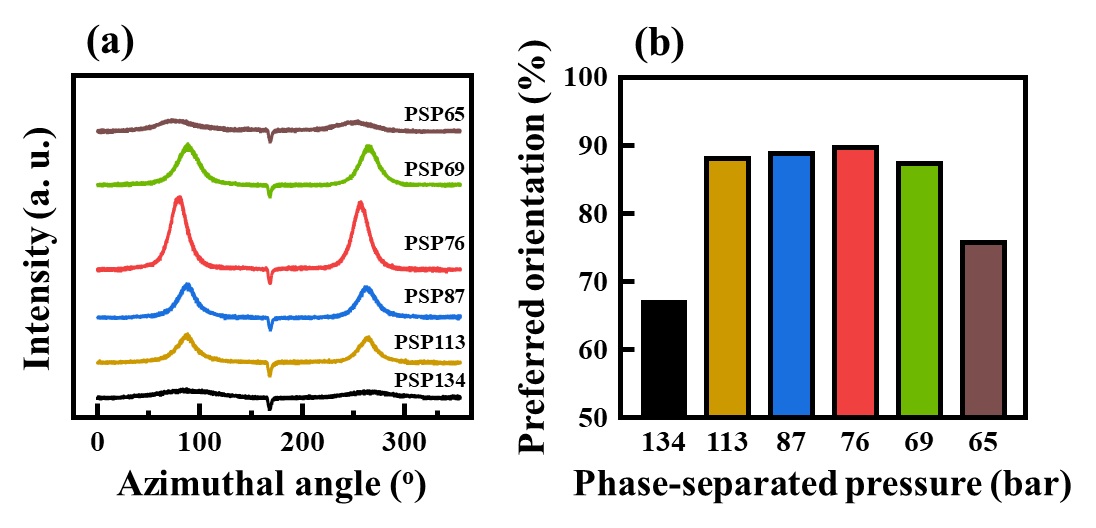
**

Figure S1. (a) Azimuthal scans at 2θ = 22° and (b) preferred orientations of FSFs obtained at different PSPs obtained by wide angle X-ray diffraction.

**
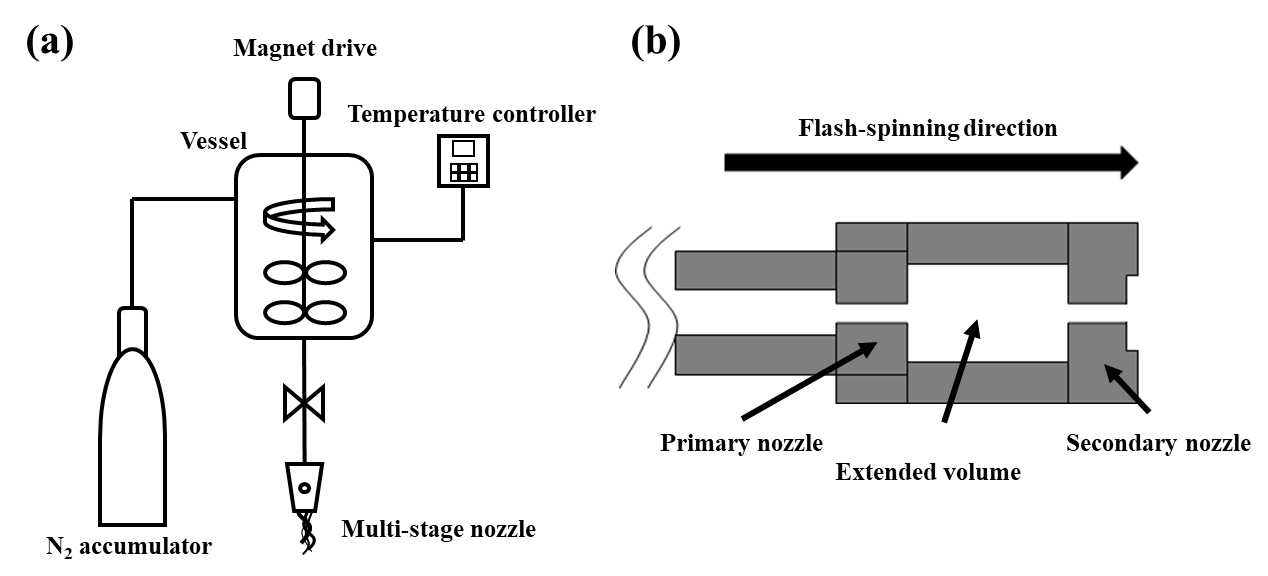
**

Figure S2. Schematics of (a) lab-scale flash-spinning apparatus and (b) multi-stage nozzle with extended volume for pressure-induced phase separation (orifice diameter is 0.7 μm).

**
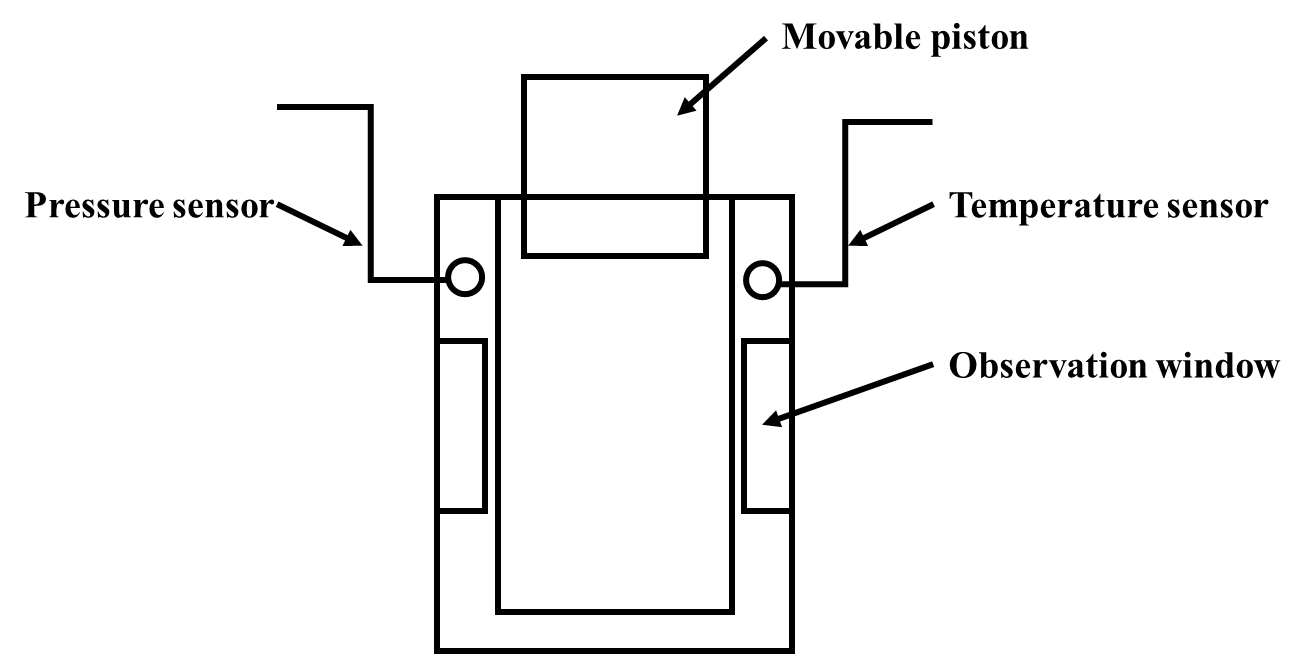
**

Figure S3. Schematic of the high-pressure view cell system.
